# The Value of MR-DWI and T1 Mapping in Indicating Radiation-Induced Soft Tissue Injury

**DOI:** 10.3389/fonc.2021.651637

**Published:** 2021-05-27

**Authors:** Zeng Wang, Bowen Xiong, Nannan Kang, Xiaoxian Pan, Caihong Wang, Li Su, Zhen Xing, Jinsheng Hong

**Affiliations:** ^1^ Central Laboratory, Cancer Center, The First Affiliated Hospital of Fujian Medical University, Fuzhou, China; ^2^ Key Laboratory of Radiation Biology of Fujian Higher Education Institutions, The First Affiliated Hospital, Fujian Medical University, Fuzhou, China; ^3^ Fujian Provincial Key Laboratory of Precision Medicine for Cancer, The First Affiliated Hospital of Fujian Medical University, Fuzhou, China; ^4^ National Health Commission Key Laboratory of Personalized Diagnosis and Treatment of Nasopharyngeal Carcinoma, Jiangxi Cancer Hospital of Nanchang University, Nanchang, China; ^5^ Department of Radiation Oncology, Jiangxi Cancer Hospital of Nanchang University, Nanchang, China; ^6^ Department of Radiology, Zhongshan Hospital Affiliated to Xiamen University, Xiamen, China; ^7^ Department of Radiotherapy, Cancer Center, The First Affiliated Hospital of Fujian Medical University, Fuzhou, China; ^8^ Department of Radiology, The First Affiliated Hospital of Fujian Medical University, Fuzhou, China

**Keywords:** magnetic resonance diffusion imaging, quantification of longitudinal relaxation time, radiation injury, soft tissue fibrosis, radiofibrosis

## Abstract

**Objective:**

To explore the value of MR-DWI and T1 mapping in predicting radiation-induced soft tissue fibrosis and its correlation with radiation inflammation.

**Methods:**

① a total of 30 C57BL/6 mice were randomly divided into a control group (Nor group), irradiation group (IR group) and irradiation plus glycyrrhetinic acid group (GA group). The IR group and GA group were treated with 6MV X-rays to irradiate the right hind limbs of mice for 30 Gy in a single shot. MRI examinations were performed before and on the 7th day after irradiation to measure the apparent diffusion coefficient (ADC) value and the longitudinal relaxation time (T1) value of the hind limb muscles of the mice. On the 90th day after irradiation, the hind limb contracture was measured, and the right hind limb muscle was taken for HE staining, masson staining, immunohistochemical staining and Western blot analysis to detect the expression of a-SMA and Fibronectin. ② The other 30 mice were grouped randomly as above. On the 7th day after irradiation, the right hind limbs of the mice were examined by MRI to measure the ADC value and T1 value of the thigh muscles, and then the right hind thigh muscles were immediately sacrificed to detect IL-1β, IL-6, TNF-a and TGF-β1 expression with ELISA.

**Results:**

On the 7th day after irradiation, the ADC values ​​of right hind thigh muscles of mice in Nor group, IR group and GA group were (1.35 ± 0.11)*10^-3^mm2/s, (1.48 ± 0.07) *10^-3^mm2/s and (1.36 ± 0.13)*10^-3^mm2/s, respectively, by which the differences between the IR group and Nor group (*P*=0.008) and that between IR group and GA group (*P*=0.013) were statistically significant; T1 values ​​were (1369.7 ± 62.7)ms, (1483.7 ± 127.7)ms and (1304.1 ± 82.3)ms, respectively, with which the differences in the T1 value between the IR group and Nor group (*P*=0.012) and between IR group and GA group (*P*<0.001) were also statistically significant. On the 90th day after irradiation, the contracture lengths of the right hind limbs of the three groups of mice were (0.00 ± 0.07)cm, (2.08 ± 0.32)cm, and (1.49 ± 0.70) cm, respectively. There were statistically significant differences in the IR group compared with the Nor group (*P*<0.001) and the GA group (*P*=0.030). The ADC value (r=0.379, *P*=0.039) and T1 value (r=0.377, *P*=0.040) of the mice’s hindlimbs on Day 7 after irradiation were correlated with the degree of contracture on Day 90 after irradiation; the ADC value (r=0.496, *P*=0.036) and T1 value (r=0.52, *P*=0.027) were positively correlated with the Masson staining results and with the expression of α-SMA and Fibronectin. While the ADC value was positively correlated with IL-6 (r=0.553, *P*=0.002), there was no obvious correlation with IL-1β, TNF-a and TGF-β1; the T1 value was positively correlated with IL-1β (r=0.419, *P*=0.021), IL-6 (r=0.535, *P*=0.002) and TNF-a (r=0.540, *P*=0.002) but not significantly related to TGF-β1 (r=0.155, *P*=0.413).

**Conclusion:**

The MR-DWI and T1 mapping values on the 7th day after irradiation can reflect the early condition of tissue inflammation after the soft tissue is irradiated, and the values have a certain correlation with the degree of radiofibrosis of the soft tissue in the later period and may be used as an index to predict radiofibrosis.

## Introduction

Cancer treatments mainly involve surgery, radiotherapy and chemotherapy. Nearly 70% of the patients with malignant tumors need to receive radiotherapy. While radiotherapy kills tumor cells, it also induces soft tissue damage. In the early stage of the injury, it is manifested as reversible acute dermatitis. Acute dermatitis after radiotherapy is the result of a combination of direct tissue damage and local inflammation. The rays cause increased vascular permeability and persistent leukocyte infiltration, leading to epidermal degeneration and dermal edema. At the same time, a large number of inflammatory factors (such as IL1, IL6 and TNF-α) are released, promoting the development of dermatitis (1). In severe cases, soft tissue ulcers or necrosis may occur, which can lead to the interruption of radiotherapy and affect the local control rate of the tumor and the survival rate of the patient. While in the late stage, as the skin continues to repair, a large number of cytokines such as TGF-β will be secreted, which will promote the activation of fibroblasts into myofibroblasts, accumulate matrix, promote scar formation, tissue contracture and eventually lead to irreversible soft tissue fibrosis. If there is a method that can predict the occurrence of fibrosis after irradiation in the early stage (i.e., the inflammation stage), timely intervention can be made in the inflammation stage to alleviate the irreversible fibrosis in the later stage (2, 3).

In recent years, with better technology developed, imaging parameters from CT and MRI have been reported to be effectively used in diagnosis, monitoring treatment response, and differential diagnosis of tumor recurrence or radiation injury (4). Magnetic resonance diffusion-weighted imaging (MR-DWI) provides quantitative parameters—an apparent diffusion coefficient (ADC) that can quantitatively reflect changes in the microstructure and function of tissues and organs. Studies have shown that the change of ADC value may be a sensitive indicator to predict the early response of breast cancer liver metastasis chemotherapy (5) and predict the local recurrence of rectal cancer (6). The study of the feasibility of the ADC-based radioimmunology model for predicting pelvic lymph node metastasis in patients with stage IB-IIA cervical squamous cell carcinoma shows that the radioimmunology model is a non-invasive preoperative prediction tool, which may have a higher predictive effect than clinical and radiological factors (7). Our previous studies have found that the ADC value changes after early tumor irradiation on the animal model of nasopharyngeal carcinoma xenograft tumor in nude mice are related to the tumor growth delay time. At the same time, the ADC value change of the early tumor radiotherapy and chemotherapy has been found to be related to short-term treatment efficacy in patients with nasopharyngeal carcinoma (8). T1 mapping is a new magnetic resonance technique. Studies have shown that T1 mapping can detect the severity of acute kidney damage in mice and predict the outcome. T1 value can reflect the water content of inflammatory tissue in the acute phase of kidney disease and the tissue fibrosis degree in its chronic phase (9). We speculate that the changes in MR-DWI and T1 mapping parameters after early irradiation can reflect the early inflammatory changes after soft tissue receiving irradiation, and may have a certain correlation with later fibrosis, and it may thus be an effective method to predict irradiation-induced soft tissue fibrosis. There are no reports on the application of MR-DWI and T1 mapping in the early prediction of irradiation-induced soft tissue fibrosis. This study intends to explore a non-invasive method to predict irradiation-induced soft tissue fibrosis based on MR-DWI and T1 mapping, so as to provide new ideas for the early prediction and prevention of irradiation-induced soft tissue fibrosis.

## Methods and Materials

### Mice Grouping and Data Collection

Thirty C57BL/6 SPF male mice aged 8 weeks were divided into three groups by the random number table method: the normal group (Nor group), the irradiation group (IR group) and the glycyrrhetinic acid (in previous studies (10), we found that glycyrrhetinic acid can inhibit radiofibrosis) group (GA group), each consisting of 10 mice. Numbers are marked by the punched holes in the ears of the mice. Both the IR group and the GA group were given a single 30Gy irradiation to the right hind limb. In addition, the GA group was given 30mg/Kg glycyrrhetinic acid with the following dosage regimen: on the day before irradiation, the day irradiation given, and the 5 days in succession after irradiation, a dose was given once a day, seven times/7 days, and then a dose was given every other day, seven times/14 days. While the Nor group and IR group were given the same dose of sterile water. MRI examination was performed before irradiation and on the 7th day after irradiation to measure ADC value and T1 value. The hind limb dermatitis of mice was observed in the early stage after irradiation (10 to 45 days after irradiation), and scores were given, at least seven times. During the late period (on the 90th day after irradiation), the right hind limb contracture was measured, and the mice were sacrificed to obtain the right hind limb thigh muscle for further related examination.

Another 30 mice were grouped and processed in the same manner as above, and MRI was performed on the 7th day after irradiation. After the inspection, the mice were sacrificed and the right hind limb muscles were taken. ELISA was employed to detect the expression of IL-1β, IL-6, TNF-a and TGF-β1. The dosage regimen, anesthesia, irradiation method for the first 7 days of this experiment and the MRI examination method, coil model, parameter, detection index and method on the 7th day after irradiation of GA group and IR group were the same as the above experiment.

#### Irradiation Method

**Figure 1:**
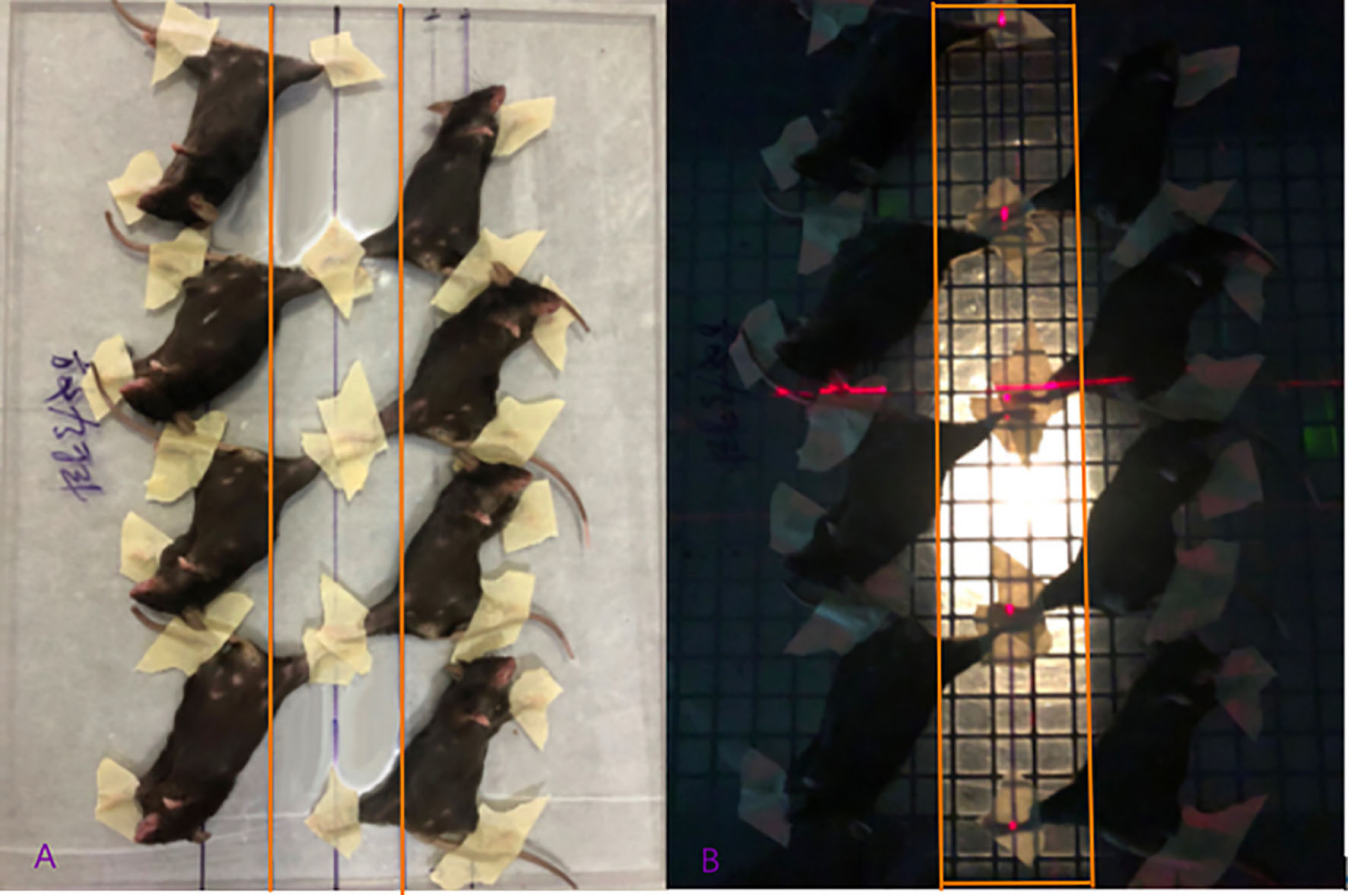
Diagram of mice fixation and the irradiation field displayed. **(A)** The mice were fixed on the glass plate two rows paralleled and opposite; **(B)** The right hind limbs of the mice were exposed to the irradiation field.

(1) Fixation method: After all mice were anesthetized, they were fixed in a supine position on a tissue compensation material (plexiglass) with a thickness of 1.0 cm. The mice were placed in a row so that the right groin lines of the mice were kept in a line, with the right hind limbs positioned toward the inside and the rest of the bodies on the other side of the line. The limbs and tails were fixed on the glass plate with tape. The contralateral mice were positioned with the same fixing method, and thus the right hind limbs of the mice on both sides of the central line were opposite (see **Figure 1A**), and eight mice can be irradiated at a time.

(2) Irradiation field size and positioning method: the field angle was 180°, the source skin distance (SSD) was 1m, and the irradiation site was on the right hind limb. The field size was 38cm×6cm (see **Figure 1B**). When set up, the bed surface was parallel to the laser light, and the control group was given the same anesthesia and fixed with the same position, and false irradiation was given on the irradiation bed.

(3) Determination of irradiation dose: a linear accelerator (Clinac600C/D) was used to irradiate the right hind limbs of mice for a single irradiation of 30Gy with 6MV-X rays at a dose rate of 200cGy/min.

#### MRI Examination Method

**Figure 2:**
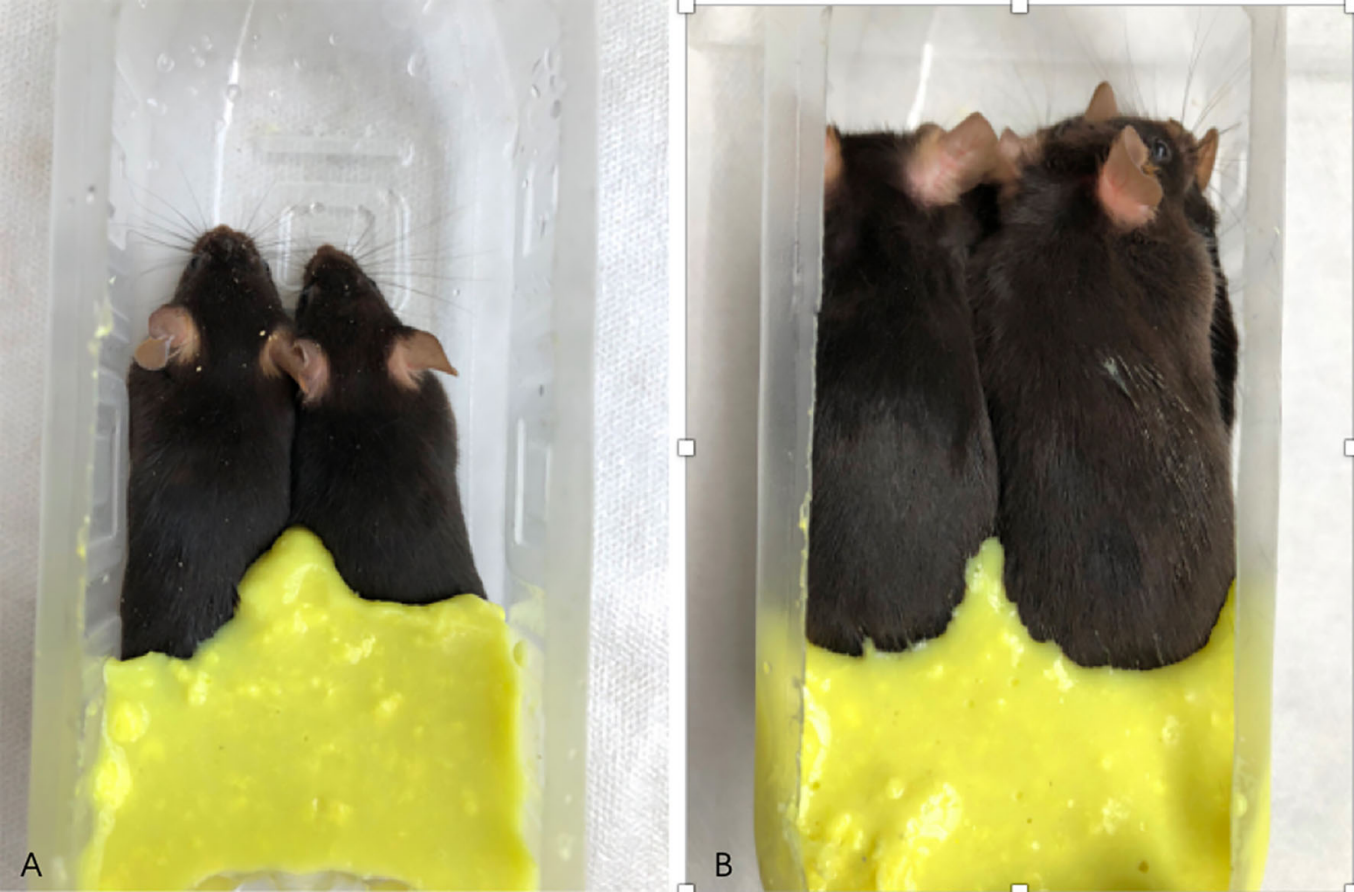
Diagram of mice being fixed and prepared for MRI examination. **(A)** shows two mice placed in a self-made container; **(B)** shows six mice stacked in a self-made container, and the gap between the hind limbs and the abdomen is filled with dental alginate printing film material.

MRI examination method: After the mice were anesthetized, two mice were placed in a prone position in parallel and in the same direction in a self-made container, with the tails at the bottom of the container, and then an appropriate amount of dental alginate printing film material (from Beijing Hong ye Dental Medical Equipment Factory) with water added and stirred into cream already was filled into the gap between the abdomens and hind limbs of the mice until it was flat at the highest point of the back of the mice. The alginate would solidify in 2–3 minutes. Then the other two mice could be placed in a prone position in turn on the back of the first two mice with the cream alginate material processed and filled in the same way as before. A total of five or six mice can be stacked (see **Figure 2**).

MRI inspection coil model and parameters: a joint surface coil was adopted, and the parameters were set as follows. Diffusion weighted imaging (DWI) series: Diffusion gradient factor (b) = (0,400) s/mm2, echo time (TE) = 47 ms, repetition time (TR) = 4490 ms, number of excitations (Nex) = 1, Matrix 116×116, scanning field of view (FOV)=134mm×134mm; Longitudinal relaxation time quantitative (T1-Mapping) sequence: echo time (TE)=2.4ms, repetition time (TR)=6.64ms, The number of excitations (Nex)=1, the matrix (Matrix) is 26×32, and the scanning field of view (FOV)=80mm×80mm.

#### MRI Detection Index and Measurement Method

**Figure 3:**
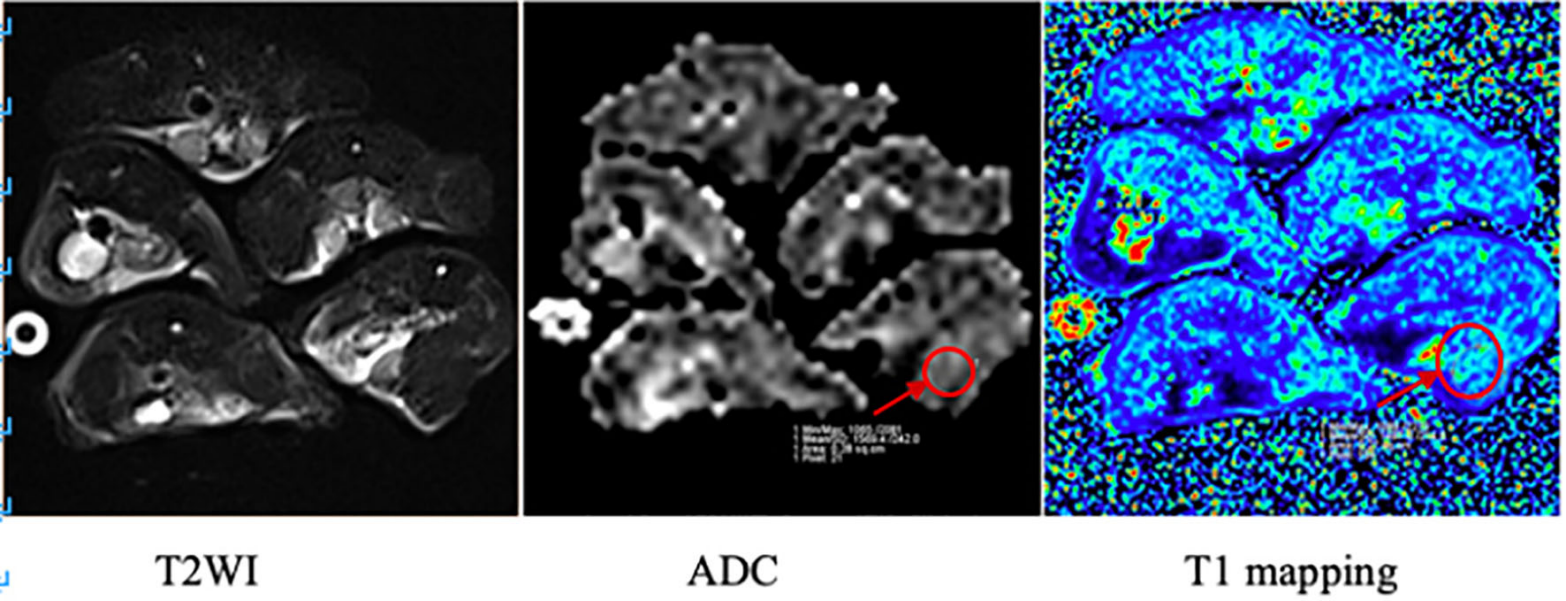
MRI images of mice in the irradiation group. The arrow in the figure shows the region of interest (ROI) of the right hind limb thigh muscle in which ADC value and T1 value were measured respectively.

The apparent diffusion coefficient (ADC) value and longitudinal relaxation time (T1) value of the upper segment of the thigh muscles on both sides of the hind limbs were measured three times, and the average value was taken (see **Figure 3**).

#### Radiation-Induced Mouse Dermatitis Score

In the acute phase of radiation dermatitis after irradiation on mice, the hair loss and skin inflammation of the right hind limbs of the mice were observed and scored.

#### Score Criteria for Mouse Skin Condition

1 point for normal skin.

1.5 points for Mild edema.

2 points for Obvious edema accompanied by hair loss, hair loss area ≤25%.

2.5 points for Depilation area>25%, but ≤75%, or accompanied by dry peeling.

3 points for Dry peeling, depilation area>75%.

3.5 points for Moist peeling, depilation area ≤25%.

4 points for Moist peeling, depilation area> 25%, but ≤ 50%.

4.5 points for Moist peeling, depilation area>50%, with a small amount of necrosis.

5 points for Large areas of skin necrosis, visible subcutaneous tissue.

#### Measurement Method of Hind Limb Contracture in the Late Stage of Irradiation

**Figure 4:**
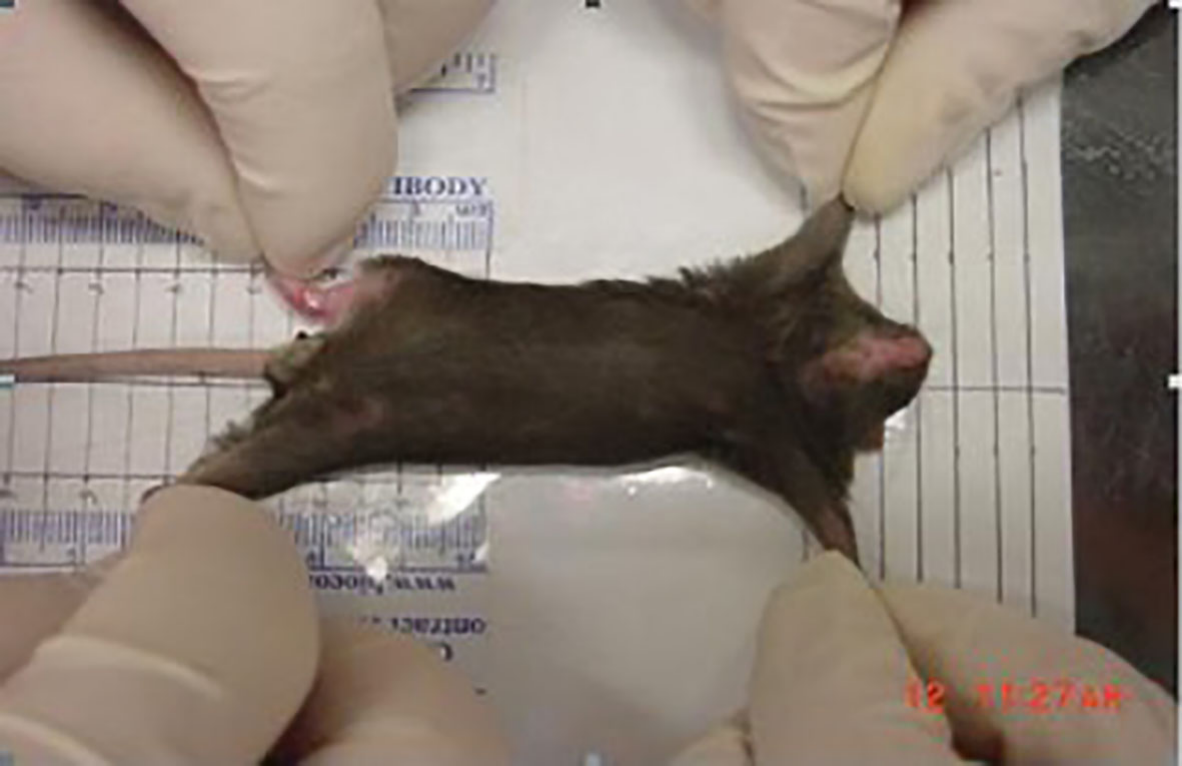
Measuring the degree of contracture of the right hind limb of irradiated mice.

The observation index of mouse soft tissue fibrosis (the degree of right hind limb contracture) is the difference between the length of the irradiated right hind limb and the normal left hind limb. The mouse was fixed on a specially designed quantitative standard ruler and kept parallel to the ruler. With the mouse’s ankle joint position as the positioning center, the ankles of both legs were held and gently pulled down at the same time. When the corresponding resistance increases greatly, stretching stopped and the distance between the heel extension point of the right hind limb ankle joint and the heel position of the left hind limb ankle joint was measured and defined as the difference between the irradiated right hind limb and the normal left hind limb (see **Figure 4**).

#### HE Staining and Masson Staining

After the muscle tissue was taken out, it was fixed with 4% paraformaldehyde for 24 h, and the sections were embedded in conventional paraffin and dehydrated. The paraffin-embedded specimens were sliced ​​continuously at a thickness of 4 μm and dried at 60°C. These slices were dewaxed twice with the xylene solution, for 30 minutes each time, and then immersed in ethanol of different concentrations before being placed in distilled water. HE and Masson staining were carried out following the kit instructions. In Masson staining, collagen fiber intensity bundles shown in blue were analyzed by Image J Program (11).

#### Measurement of Cytokines With ELISA

Tissue samples from mice were homogenized in lysis buffer (50 mM Tris-HCl buffer pH 8.0 with 120 mM NaCl, 1 mM EDTA, 6 mM EGTA, 1% NP-40 and 1 mM dithiothreitol) supplemented with phosphatase inhibitors and protease inhibitor (Sangon Biotech, China). The lysis product was centrifuged at 12000g for 3 min, and the supernatant was taken for protein quantification according to the manufacturer’s instructions of the BCA quantification kit (Thermo Fisher Scientific, USA), and all the sample protein concentrations were adjusted to 2 mg/ml with lysis buffer. Mouse IL-1β, IL-6, TNF-a and TGF-β1 levels in the extracts were measured according to the manufacturer’s instructions, using specific ELISA kits (MultiSciences Biotech, China) respectively. Three replicate wells were set up for each sample and the results were expressed in pg/mg.

#### Immunohistochemistry

Tissue sections with a thickness of 4μm were deparaffinized, rehydrated, and immersed in sodium citrate buffer to restore the antigen. Then, 3% H_2_O_2_ was used to block endogenous peroxidase for 10 minutes, followed by blocking with 5% bovine serum albumin (BSA) for 30 minutes. The sections were incubated with anti-α-SMA (1:500; 19245, CST Germany) and anti-Fibronectin (1:800; ab2413, Abcam USA) antibodies at 4°C overnight. The sections were washed with PBS buffer three times and placed with the secondary antibody coupled with horseradish peroxidase for 1 h at room temperature. DAB was then used to develop color. Finally, hematoxylin was employed for counterstaining. After dehydration and transparency, they were observed and picture taken under a microscope. Three random fields of view were taken for each sample, and the average optical density was calculated by Image J Program.

#### Western Blot

The total protein was extracted with RIPA buffer, and the protein concentration was determined by using a BCA kit. After the protein samples were subjected to polyacrylamide gel electrophoresis, they were transferred to a polyvinylidene fluoride membrane, sealed with 5% skimmed milk powder at room temperature for 1 h, and then the primary antibody (1:1 000) was added separately, incubated overnight at 4°C. Then, the membrane was washed with TBST before secondary antibody (1: 2 000) was added and incubated at room temperature for 90 min, again washed with TBST, and developed with ECL luminescent solution. The imaging system was used to examine and the gray value of each histone band could be determined by Image J software. The relative protein expression level = target band Gray value/β-actin band gray value. There are three replicates for each protein sample.

## Statistical Analysis

Data were analyzed with SPSS 19.0 statistical software, and the normality test was further confirmed by the K-S method and double-checked by the Q-Q graphic method. The homogeneity of variance test was performed by the F test. When the samples were normally distributed and the variances were uniform, the three groups were compared by analysis of variance, and the two independent sample means were compared with t test. When the samples did not meet the above conditions, the three groups were compared with the Kruskal-Wallis nonparametric test, and the two groups were compared with t test or Mann-Whitney U rank sum test. Image-Pro Plus and Image J were used for analysis and statistics of IHC image and Western blot bands, and Graphpad Prism 6 software was used for drawing. *P*<0.05 means the difference is statistically significant.

## Result

### Radiation-Induced Dermatitis in Mice

**Figure 5:**
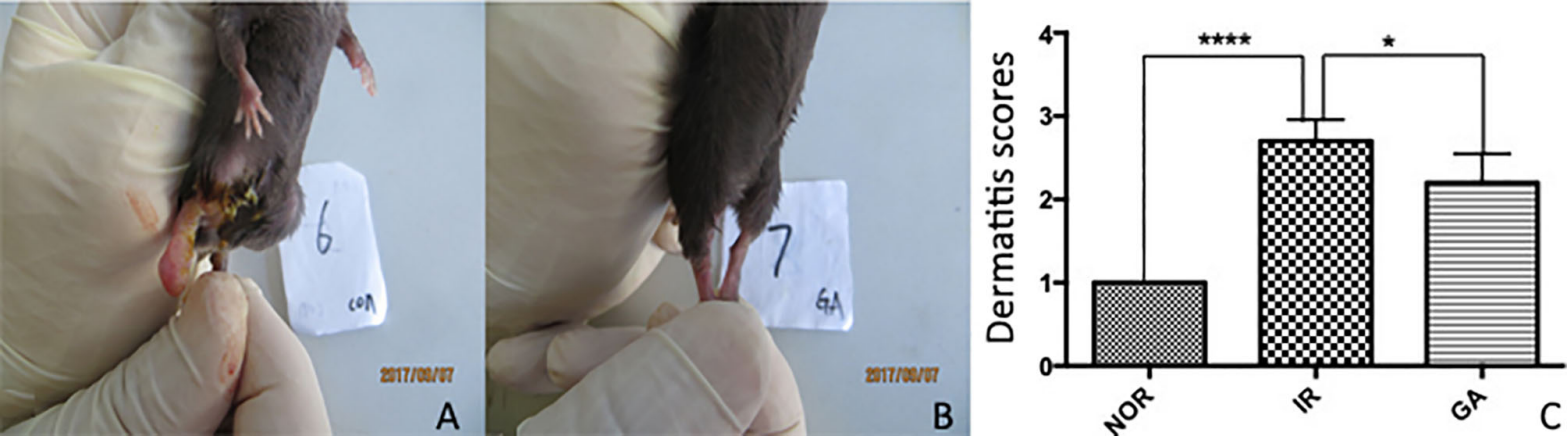
The dermatitis state and scores of the right hind limbs of mice on the 19th day after irradiation. **(A, B)** The dermatitis state of the IR group No. 6 mouse and the GA group No. 7 mouse, respectively. **(C)** The comparison between the dermatitis scores of each group. * means P<0.05, **** means P<0.0001, Nor refers to normal group, IR simple irradiation group, GA GA group.

Radiation dermatitis was observed in both the IR group and the GA group after irradiation, and the state of the IR group was significantly worse than that of the GA group (see **Figure 5**). On the 19th day after irradiation, the dermatitis scores of the Nor group, IR group, and GA group were (1.00 ± 0.00), (2.70 ± 0.26), (2.20 ± 0.35) points respectively. The Kruskal-Wallis nonparametric test was used, and the result was as follows: H=24.468, *P*<0.0001, where the differences between the IR group and Nor group (*P*<0.0001) and the IR group and GA group (*P*=0.005) were statistically significant.

### Irradiation Caused an Increase of Inflammatory Factors

**Figure 6:**
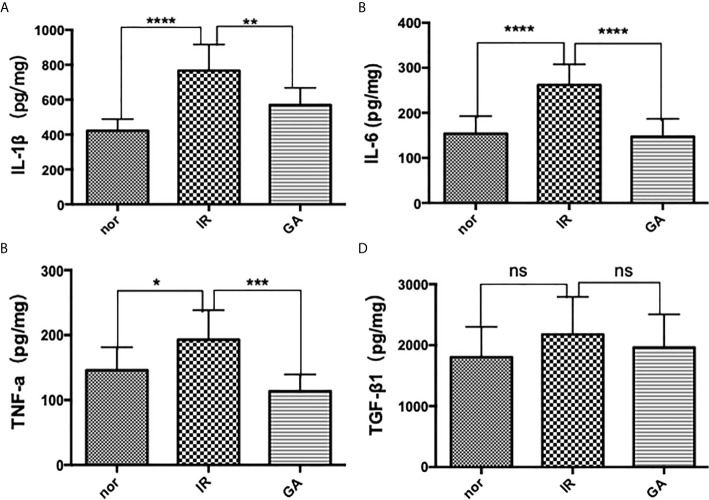
The expressions of IL-1β, IL-6, TNF-a and TGF-β1 in the muscles of the right hind limbs of the mice were detected by ELISA on the 7th day after irradiation. **(A–D)** are the expression graphs of IL-1β, IL-6, TNF-a and TGF-β1, respectively; ns means *P*>0.05, * means *P*<0.05, ** means *P*<0.01, *** means *P*<0.001, **** means *P*<0.0001.

ELISA was used to detect the expression of IL-1β, IL-6, TNF-a and TGF-β1 in the right hind limb muscles of mice on the 7th day after irradiation. The expressions of IL-1β in Nor group, IR group and GA group were as follows: (422.1 ± 66.5)pg/mg, (766.1 ± 150.2)pg/mg and (569.7 ± 97.6)pg/mg, respectively; those of IL-6 were (153.8 ± 39.1)pg/mg, (261.9 ± 45.8)pg/mg and (147.0 ± 39.8)pg/mg, respectively; and those of TNF-a were (145.8 ± 35.6)pg/mg, (192.8 ± 45.5)pg/mg and (113.6 ± 25.7)pg/mg, respectively. In the IR group compared with Nor group and GA group, the expressions of all the inflammatory factors were significantly increased. However, the expressions of TGF-β1 in the Nor group, IR group and GA group were (1804.6 ± 496.0) pg/mg, (2176.0 ± 617.8) pg/mg and (1965.0 ± 541.9) pg, respectively, and there were no significant differences between the IR group and Nor group (*P*=0.146) or between IR group and GA group (*P*=0.402) (see **Figure 6**).

### Irradiation Induced Hindlimb Contracture

**Figure 7:**
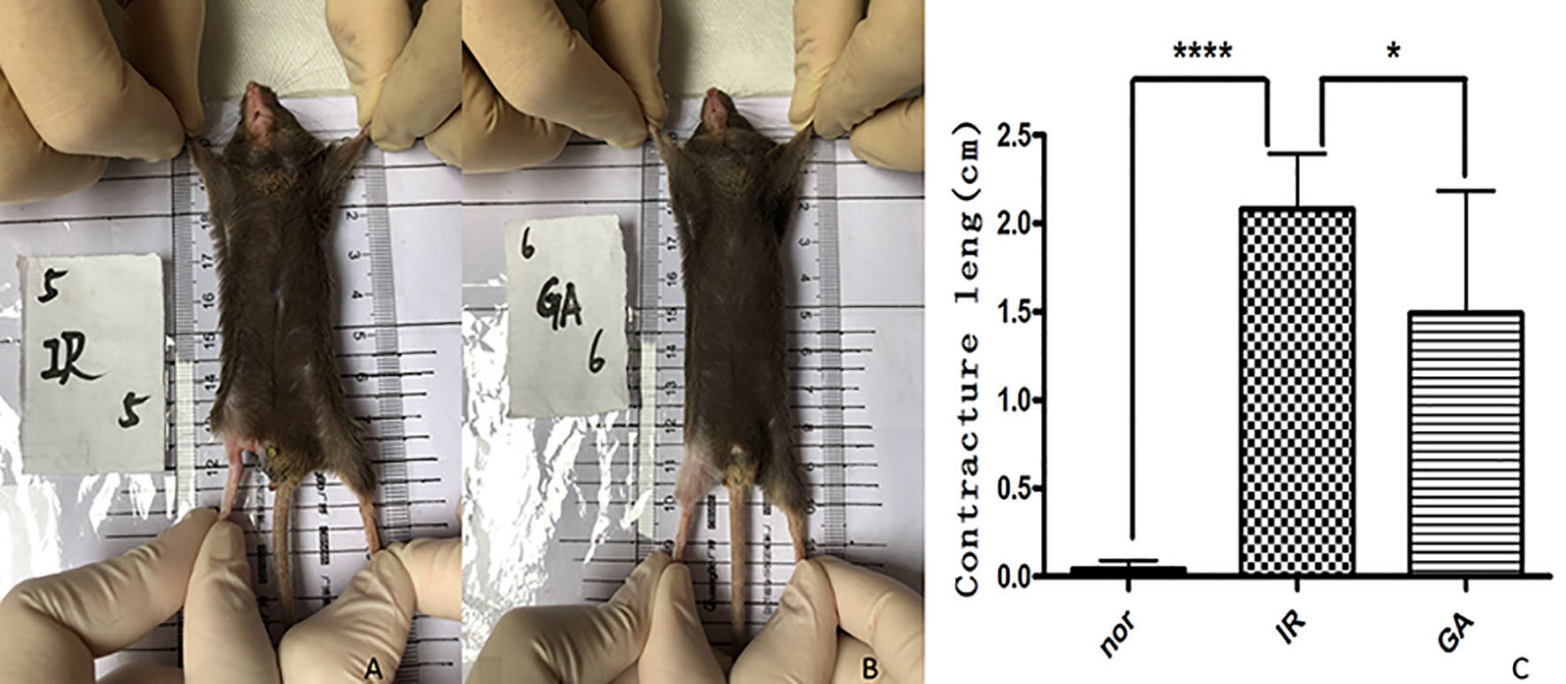
Contracture of the right hind limbs of the mice in the three groups on the 90th day after irradiation. **(A, B)** are mice in the IR group and GA group respectively, and **(C)** is the statistics of contracture length in each group. * means *P*<0.05, **** means *P*<0.0001.

After 1.5 months of irradiation, it was observed that the right hind limbs of some mice in the IR group and the GA group began to develop contractures, but the state of the GA group was less serious than that of the IR group. The contractures were measured on the 90th day after irradiation (see **Figure 7**). The contracture lengths of the right hind limbs of the mice in the Nor group, IR group and GA group were (0.00 ± 0.07) cm, (2.08 ± 0.32) cm and (1.49 ± 0.70) cm, respectively. The differences between IR group and Nor group (P<0.0001) and between IR group and GA group (P=0.030) were statistically significant.

### Irradiation Caused Muscle Fibrosis

**Figure 8:**
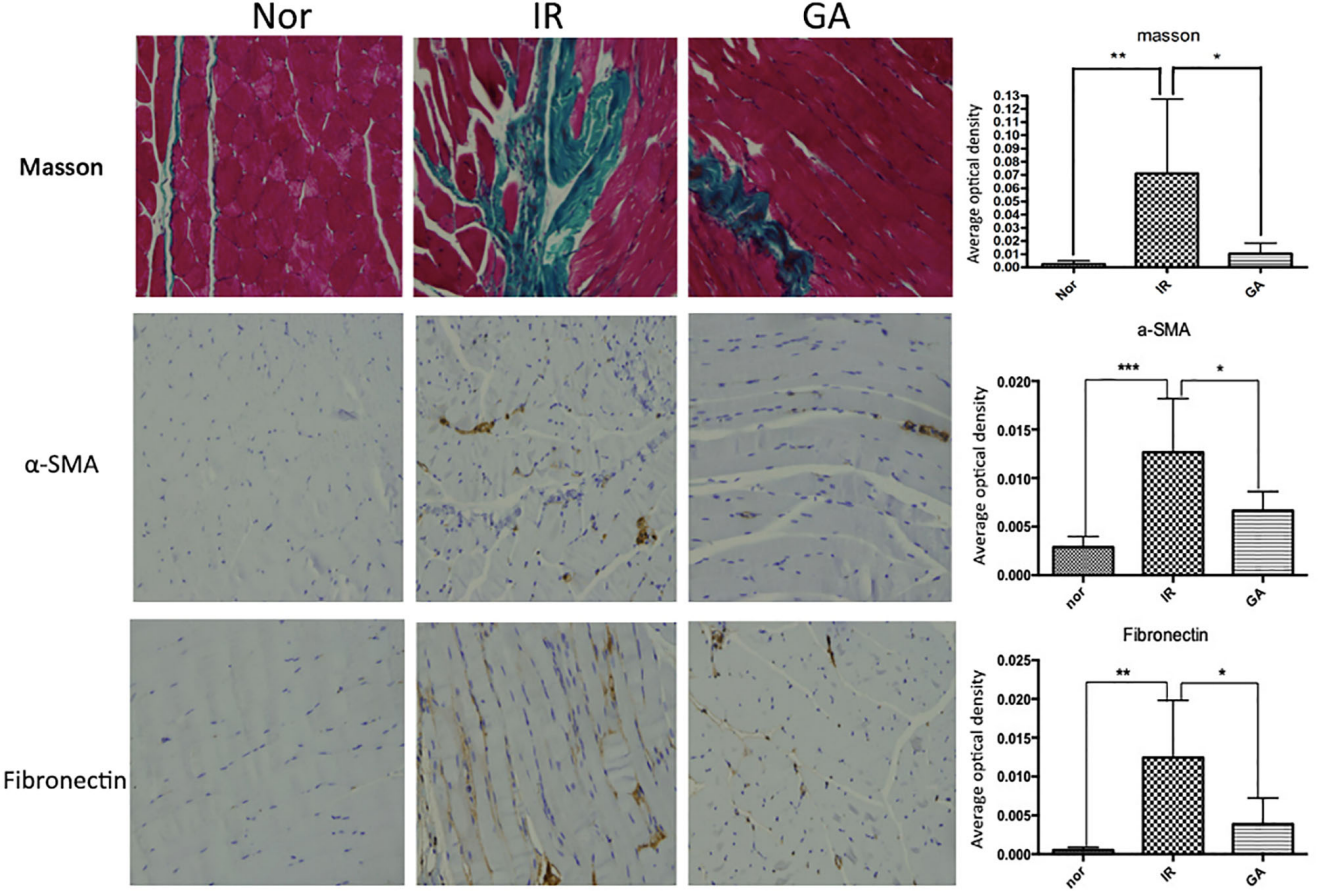
Masson staining and IHC detection of the expressions of α-SMA, fibronectin in the right hind thigh muscles on the paraffin sections (200×) and statistics of the average optical density of each group on the 90th day after irradiation. * means *P*<0.05, ** means *P*<0.01, *** means *P*<0.001.

On the 90th day after irradiation, HE staining of the right hind limbs of the IR group and GA group showed that the muscle fibers of the mice in these two groups were more disorderly than those of mice in the Nor group, and the collagen fibers stained in dark red were seen, which was more obvious in the IR group than in the GA group. Masson staining showed that the collagen fibers dyed in green were deposited in the muscle fiber gaps in the IR group and the GA group. The average optical density values ​​of Masson staining in the Nor group, IR group and GA group were 0.003 ± 0.002, 0.071 ± 0.056 and 0.010 ± 0.008, respectively. There were statistically significant differences between the IR group and Nor group (*P*=0.031) and between the IR group and GA group (*P*=0.046) (see **Figure 8**).

The immunohistochemical wax sections were observed under the microscope, the expression of α-SMA and fibronectin in the right hind thigh muscles of the mice in the IR group were higher than those of the mice in the Nor group on the 90th day after irradiation, while the expression levels in the GA group was lower when compared with the IR group. When α-SMA was concerned, the average optical density values ​​of α-SMA in Nor group, IR group and GA group were 0.003 ± 0.001, 0.013 ± 0.006 and 0.007 ± 0.002, respectively. Compared with the Nor group (*P*<0.001) and with the GA group (*P*=0.009), the differences in the IR group were statistically significant. When fibronectin was concerned, the average optical density values ​​of fribronectin in the Nor group, IR group and GA group are 0.001 ± 0.000, 0.013 ± 0.007 and 0.004 ± 0.003, respectively. The differences between the IR group and Nor group (*P*=0.011) and between the IR group and GA group (*P*=0.027) were statistically significant (see **Figure 8**).

Western blot method was also used to detect the expression quantity of α-SMA and fibronectin in the thigh muscles of the right hind limbs of the three groups of mice. The expression of α-SMA and fibronectin in the Nor group was very low. Both the IR and GA groups showed different levels of expression, but the level of the GA group was significantly lower than that of the IR group. When α-SMA is concerned, the average optical density values of the ratio of α-SMA gray value of the Nor group, IR group and GA group to the gray value of respective internal reference β-actin (α-SMA/β-actin) ​​were 0.537 ± 0.095, 1.271 ± 0.236 and 0.666 ± 0.157, respectively. The IR group was significantly different from the Nor group (*P*<0.001) and from the GA group (*P*=0.001). When fibronectin is concerned, the average optical density values of the ratio of the fibronectin gray value of the Nor group, IR group and GA group were 0.078 ± 0.013, 1.521 ± 0.376 and 0.269 ± 0.226, respectively. There were statistically significant differences between the IR group and the Nor group (*P*<0.0001) and between the IR group and the GA group (*P*=0.0001).

### Irradiation Caused an Increase in ADC Value and T1 Value

The ADC values of the right hind thigh muscles of the three groups of mice before irradiation were (1.31 ± 0.07)×10^-3^mm2/s, (1.32 ± 0.08)×10^-3^mm2/s and (1.32 ± 0.07)×10^-3^mm2/s, and the T1 values were (1381.7 ± 41.4) ms, (1388.9 ± 69.2) ms and (1386.0 ± 65.7) ms, respectively. There was no statistically significant difference among the three groups. On the 7th day after irradiation, the ADC values of the right hind limb muscles of the three groups of mice were (1.35 ± 0.11)×10^-3^mm2/s, (1.48 ± 0.07)×10^-3^mm2/s and (1.36 ± 0.13)×10^-3^mm2/s, with the ADC value of the IR group higher than that of the Nor group (P=0.008) and the GA group (P=0.013); and the T1 values of the right hind thigh muscles of the mice in the Nor group, IR group and GA group were (1369.7 ± 62.7)ms, (1483.7 ± 127.7)ms and (1304.1 ± 82.3)ms, respectively, with a higher T1 value in the IR group, compared with that of Nor group (*P*=0.012) and with that of GA group (*P*<0.001).

### Correlation Between ADC, T1 Value and Inflammation After Irradiation

On the 7th day after irradiation, the ADC value of the right hind limb muscles of the mice was positively correlated with IL-6 (r=0.553, *P*=0.002), but there were no significant correlations between ADC and IL-1β and between ADC and TNF-a. And T1 value is positively correlated with IL-1β (r=0.419, *P*=0.021), IL-6 (r=0.535, *P*=0.002) and TNF-a (r=0.540, *P*=0.002).

Correlation between ADC, T1 value and fibrosis in the late period after irradiation.

The ADC value (correlation coefficient r=0.379, *P*=0.039) and T1 value (correlation coefficient r=0.377, *P*=0.040) of the hindlimbs of the mice on the 7th day after irradiation were positively correlated with the degree of contracture on the 90th day after irradiation. The ADC value (r=0.496, *P*=0.036) and T1 value (r=0.52, *P*=0.027) were positively correlated with the Masson staining results. The was also a positive correlation between the ADC value (r=0.516, *P*=0.028; r=0.559, *p*=0.016), T1 value (r=0.655, *p*=0.003; r=0.551, *p*=0.018) and the immunohistochemical detected α-SMA and fibronectin expressions. The results of Western blot detection also showed that the ADC value (r=0.582, *p*=0.047; r=0.574, *p*=0.051), T1 value (r=0.773, *p*=0.003; r=0.792, *p*=0.002) and α- SMA and fibronectin protein expressions were positively correlated.

## Discussion

The results of this study found that the changes in ADC and T1 values ​​detected in the early stage of irradiation in mice were correlated with soft tissue fibrosis in the later stage of irradiation, and the expression levels of inflammatory factors in the early stage of soft tissue irradiation were also correlated with later soft tissue fibrosis. This study verified this correlation from several aspects, such as leg contracture length, pathological changes and molecules. Studies have shown that ADC and T1 values ​​can not only reflect inflammation but also the degree of soft tissue fibrosis induced by radiotherapy.

In recent years, the imaging parameters of MRI are often used to diagnose and differentiate tumors. By using advanced imaging techniques, such as magnetic resonance imaging (MRI), computed tomography (CT), SPECT and PET radioisotope research, tumor-related facts, such as blood vessel contour, water content, degree of apoptosis, necrosis or metabolism, can be measured. Magnetic resonance diffusion-weighted imaging (MR-DWI) provides a quantitative parameter—an apparent diffusion coefficient (ADC) which size depends on the viscosity of the molecule, the permeability of the cell membrane, the direction of the tissue and the cell structure that hinders the movement of water molecules (12, 13). DWI imaging technology indirectly reflects the changes in tissue microstructure and cell function by detecting changes in the motion state of water molecules in biological tissues. Studies have shown that ADC and histological measurements of cell density in liver metastases of colorectal cancer are negatively correlated (14). A study of 32 patients with locally advanced gastroesophageal cancer showed that there is a correlation between the changes in ADC estimates after neoadjuvant therapy and the degree of tumor regression determined by histology (15).

Compared with ADC value, T1 mapping is a new magnetic resonance technique, a cardiac magnetic resonance (CMR) imaging technique, which can directly measure tissue T1 relaxation value (referred to as T1 value), reflecting the edema of myocardial cells and the degree of fibrosis of interstitium, so as to evaluate local and diffuse myocardial lesions. Prolonged myocardial T1 values ​​occur in most pathological conditions, including edema, as well as some chronic cardiac insufficiency and systemic diseases (16). There are also studies that apply T1 mapping to the evaluation of liver fibrosis and liver function, and they found that it had an important value (17, 18). Existing studies have shown that ADC changes are related to the degree of tumor regression and are capable of predicting the aggressiveness and recurrence of some tumors. The T1 value can reflect the acute injury of some organs and predict the outcome. In this study, we used ADC value and T1 value to detect the severity of radiographic inflammation in the early stages of injury, and since there is a correlation between inflammation and fibrosis, we also verified the correlation between early measured ADC/T1 value and fibrosis. There are not many studies on the application of MRI to the early damage after radiotherapy. In a study on the value of using magnetic resonance (MR) to quantitatively evaluate the early radiation damage of the salivary glands of patients with nasopharyngeal carcinoma (IMRT), the ADC value of the salivary glands at the early stage after radiotherapy for nasopharyngeal carcinoma is significantly higher than that before radiotherapy. It is believed that MRI can quantitatively evaluate the early changes of the salivary glands after IMRT radiotherapy for nasopharyngeal carcinoma and has a high potential for clinical application (19). The increase in ADC of the parotid glands after radiotherapy may be due to the loss of acinar that resulted in wider inter-cellular space, or it may be due to edema during inflammation, which is still unclear (20). The application of T1 value to early radiation damage is rarely reported. T1 mapping is currently one of the most popular means of quantitative assessment, with the advantages of high resolution, short imaging time and insensitivity to artifacts compared to ADC maps. T1 mapping reflects the change in the longitudinal relaxation time of the tissue, which is mainly influenced by the proportion of water in the tissue. Whereas ADC mainly responds to the density of cells in the region. In this study, both the ADC value and T1 value of the thigh muscles increased significantly when measured on the 7th day after the hindlimb of the mice were irradiated and the treatment group decreased. At the beginning of radiation-induced inflammation (e.g., day 7), animals begin to show relatively obvious acute radiation skin damage, with the main changes being tissue fluid exudation, congestion, edema, and inflammatory cell infiltration. These changes will significantly affect the tissue water content while changing the cell density very little, so we can find that on the 7th day after irradiation, the ADC value was positively correlated with IL-6, but there were no significant correlations between ADC and IL-1β and between ADC and TNF-a, the corresponding T1 values, however, showed a positive correlation with all three inflammatory factors. In radiation injury, TGF-β1 plays a role in promoting damage repair, and its production is later than the inflammatory response at the beginning of the injury, so we observed that the T1 value detected at day 7 is not significantly correlated with it.

The results of this study found that functional MRI may be used as a noninvasive assessment of radiotherapy-induced soft tissue injury, but it needs to be verified in further clinical studies. We plan to apply the MR-DWI and T1 mapping sequence to patients with nasopharyngeal carcinoma to observe the relationship between the changes in the ADC value and T1 value of the neck soft tissue after irradiation and the degree of soft tissue fibrosis in the later stage in the hope of getting wider ground for applying the ADC value and T1 value to predict soft tissue fibrosis after radiotherapy.

## Conclusion

We found through a mouse model that ADC and T1 values are related to radiation dermatitis and also related to radiation fibrosis, which can be used as a non-invasive means to predict the severity of radiation-induced soft tissue fibrosis in the early stage of radiation therapy.

## Data Availability Statement

The raw data supporting the conclusions of this article will be made available by the authors, without undue reservation.

## Ethics Statement

The animal study was reviewed and approved by Ethics Committee of Fujian Medical University.

## Author Contributions

JH provided the idea of this research and designed the study. BX and LS irradiated mice. NK and ZX detected mice using MRI. XP and CW did HE and IHC. BX and ZW did ELISA and WB. JH, BX and ZW wrote the manuscript. BX did the statistical analysis. All authors contributed to the article and approved the submitted version.

## Funding

This study was funded by the Training project of young talents in the health system of Fujian Province (NO.2015-ZQN-ZD-19).

## Conflict of Interest

The authors declare that the research was conducted in the absence of any commercial or financial relationships that could be construed as a potential conflict of interest.

## References

[1] Hegedus F Mathew LM Schwartz RA. Radiation Dermatitis: An Overview. Int J Dermatol (2017) 56(9):909–14. 10.1111/ijd.13371 27496623

[2] DiFrancesco T Khanna A Stubblefield MD. Clinical Evaluation and Management of Cancer Survivors With Radiation Fibrosis Syndrome. In: Seminars in Oncology Nursing. Philadelphia, United States: Elsevier (2020). 10.1016/j.soncn.2019.150982 32008860

[3] Wang B Wei J Meng L Wang H Qu C Chen X. Advances in Pathogenic Mechanisms and Management of Radiation-Induced Fibrosis. Biomed Pharmacother (2020) 121:109560. 10.1016/j.biopha.2019.109560 31739160

[4] Winfield J Payne G Desouza N. Functional MRI and CT Biomarkers in Oncology. Eur J Nucl Med Mol Imaging (2015) 42(4):562–78. 10.1007/s00259-014-2979-0 25578953

[5] Bai G Wang Y Zhu Y Guo L. Prediction of Early Response to Chemotherapy in Breast Cancer Liver Metastases by Diffusion-Weighted MR Imaging. Technol Cancer Res Treat (2019) 18:1533033819842944. 10.1177/1533033819842944 30961445 PMC6457027

[6] Elmi A Hedgire S Covarrubias D Abtahi S Hahn P Harisinghani M. Apparent Diffusion Coefficient as a non-Invasive Predictor of Treatment Response and Recurrence in Locally Advanced Rectal Cancer. Clin Radiol (2013) 68(10):e524–31. 10.1016/j.crad.2013.05.094 23830776

[7] Yu YY Zhang R Dong RT Hu QY Yu T Liu F. Feasibility of an ADC-based Radiomics Model for Predicting Pelvic Lymph Node Metastases in Patients With Stage IB–IIA Cervical Squamous Cell Carcinoma. Br J Radiol (2019) 92(1097):20180986. 10.1259/bjr.20180986 30888846 PMC6580913

[8] Hong J Yao Y Zhang Y Tang T Zhang H Bao D. Value of Magnetic Resonance Diffusion-Weighted Imaging for the Prediction of Radiosensitivity in Nasopharyngeal Carcinoma. Otolaryngol Head Neck Surg Off J Am Acad Otolaryngol Head Neck Surg (2013) 149(5):707–13. 10.1177/0194599813496537 23884282

[9] Hueper K Peperhove M Rong S Gerstenberg J Mengel M Meier M. T1-Mapping for Assessment of Ischemia-Induced Acute Kidney Injury and Prediction of Chronic Kidney Disease in Mice. Eur Radiol (2014) 24(9):2252–60. 10.1007/s00330-014-3250-6 24996794

[10] Su L Wang Z Huang F Lan R Chen X Han D. 18β-Glycyrrhetinic Acid Mitigates Radiation-Induced Skin Damage Via NADPH Oxidase/ROS/p38MAPK and NF-κb Pathways. Environ Toxicol Pharmacol (2018) 60:82–90. 10.1016/j.etap.2018.04.012 29677640

[11] Rasband W. Imagej: Image Processing and Analysis in Java. Astrophysics Source Code Library (2012) ascl:1206.013. 10.1353/aza.0.0071

[12] Razek AAKA. Routine and Advanced Diffusion Imaging Modules of the Salivary Glands. Neuroimaging Clinics (2018) 28(2):245–54. 10.1016/j.nic.2018.01.010 29622117

[13] Kauppinen RA. Monitoring Cytotoxic Tumour Treatment Response by Diffusion Magnetic Resonance Imaging and Proton Spectroscopy. NMR Biomed: Int J Devoted Dev Appl Magnet Resonance Vivo (2002) 15(1):6–17. 10.1002/nbm.742 11840548

[14] Heijmen L Ter Voert EE Nagtegaal ID Span P Bussink J Punt CJ. Diffusion-Weighted MR Imaging in Liver Metastases of Colorectal Cancer: Reproducibility and Biological Validation. Eur Radiol (2013) 23(3):748–56. 10.1007/s00330-012-2654-4 23001604

[15] De Cobelli F Giganti F Orsenigo E Cellina M Esposito A Agostini G. Apparent Diffusion Coefficient Modifications in Assessing Gastro-Oesophageal Cancer Response to Neoadjuvant Treatment: Comparison With Tumour Regression Grade At Histology. Eur Radiol (2013) 23(8):2165–74. 10.1007/s00330-013-2807-0 23588582

[16] Aherne E Chow K Carr J. Cardiac T1 Mapping: Techniques and Applications. J Magnet Resonance Imaging (2020) 51(5):1336–56. 10.1002/jmri.26866 31334899

[17] Hoffman DH Ayoola A Nickel D Han F Chandarana H Shanbhogue KP. T1 Mapping, T2 Mapping and MR Elastography of the Liver for Detection and Staging of Liver Fibrosis. Abdominal Radiol (2020) 45(3):692–700. 10.1007/s00261-019-02382-9 31875241

[18] Li J Liu H Zhang C Yang S Wang Y Chen W. Native T1 Mapping Compared to Ultrasound Elastography for Staging and Monitoring Liver Fibrosis: An Animal Study of Repeatability, Reproducibility, and Accuracy. Eur Radiol (2020) 30(1):337–45. 10.1007/s00330-019-06335-0 31338650

[19] Zhou S Qian J Xu L Tian Y Fan Q Shen J. The Quantitative Evaluation of Early Radiation-Induced Changes in the Salivary Glands Using MRI. Zhonghua yi xue za zhi (2017) 97(7):492–5. 10.3760/cma.j.issn.0376.2491.2017.07.004 28260286

[20] Juan C-J Cheng C-C Chiu S-C Jen Y-M Liu Y-J Chiu H-C. Temporal Evolution of Parotid Volume and Parotid Apparent Diffusion Coefficient in Nasopharyngeal Carcinoma Patients Treated by Intensity-Modulated Radiotherapy Investigated by Magnetic Resonance Imaging: A Pilot Study. PloS One (2015) 10(8):e0137073. 10.1371/journal.pone.0137073 26323091 PMC4556378

